# Hormonal contraception and risk of breast cancer and breast cancer in situ among Swedish women 15–34 years of age: A nationwide register-based study

**DOI:** 10.1016/j.lanepe.2022.100470

**Published:** 2022-07-29

**Authors:** Jenny Niemeyer Hultstrand, Kristina Gemzell-Danielsson, Helena Kopp Kallner, Henrik Lindman, Per Wikman, Inger Sundström-Poromaa

**Affiliations:** a Department of Women's and Children's Health, Uppsala university, SE-75185 Uppsala, Sweden; b Department of Women's and Children's Health, Karolinska institutet, SE-17177 Stockholm, Sweden; c Department of Immunology, Genetics and Pathology, Uppsala University, Rudbecklaboratoriet, SE-75185 Uppsala, Sweden

**Keywords:** Hormonal contraception, Progestagen, Progestagen-only contraception, Breast cancer, Population-based, ATC, Anatomical Therapeutic Chemical, CI, Confidence Interval, DDD, Defined Daily Dose, DMPA, Depot medroxyprogesterone acetate, ICD-10, International Classification of Diseases and Related Health Problems version 10, IRR, Incidence Rate Ratio, HC, Hormonal Contraception, LNG-IUS, Levonorgestrel intrauterine system, SPDR, Swedish Prescribed Drug Register

## Abstract

**Background:**

Evidence on a possible association between newer hormonal contraceptives (HC) and risk of breast cancer remains inconclusive, especially as concerns progestogen-only methods.

**Methods:**

In this nationwide prospective cohort study, all Swedish women aged 15–34 at study start on January 1^st^ 2005, or who turned 15 years during the study period, were followed until December 31^st^ 2017. Using information from seven National Registers, we assessed the risk ratio of developing breast cancer and breast cancer in situ in relation to different HC using Poisson regression. We adjusted the analyses for several known confounders of breast cancer.

**Findings:**

This cohort included 1.5 million women providing more than 14 million person-years. During the study period, 3842 women were diagnosed with breast cancer. Compared with never users of any HC, we found no increased risk of developing breast cancer among current users of any combined HC, IRR 1.03 (0.91–1.16), whereas current users of progestogen-only methods had an increased risk of developing breast cancer, IRR 1.32 (1.20–1.45). Across all types of HC, the risk of developing breast cancer appeared to be highest the first five years of use (combined HC IRR 1.39 (1.14–1.69); progestogen-only methods IRR 1.74 (1.44–2.10). The risk disappeared ten years after the women stopped using HC. The absolute risk of breast cancer per 100,000 women-years was 22.4 for never users, 10.9 for current users of combined HC, and 29.8 for current users of progestogen-only methods.

**Interpretation:**

Current use of progestogen-only methods is associated with a small increased risk of developing breast cancer, whereas we could only detect an increased risk among users of combined HC during the first five years of use. This may partly be explained by a selective prescription of progestogen-only methods to women with risk factors for breast cancer, like smoking or obesity. As the absolute risk of breast cancer was small, the many health benefits associated with HC must also be taken into account in contraceptive counselling.

**Funding:**

This study was funded by the Swedish Cancer Society and by the Uppsala County Council, the Faculty of Medicine at Uppsala University.

**Research in context**

**Evidence before this study**

On October 6, 2021 we searched PubMed for articles published between 1985 and 2021 without any language restrictions using the search terms (“breast cancer”) AND (“hormonal contraception” OR “hormonal contraceptives” OR "progestin" OR "progestogen").

Current use of HC has been associated with a small increased relative risk of breast cancer in several studies. This risk appears to disappear gradually within five to ten years after cessation. However, evidence on progestogen-only methods is lacking, and many studies include methods that are no longer used. New methods have arrived on the market, including lower-dose combined oral contraceptives and efficacious progestogen-only preparations.

**Added value of this study**

In this nationwide prospective cohort study, all Swedish women aged 15–34 at study start on January 1^st^ 2005, or who turned 15 years during the study period, were followed until December 31^st^ 2017, providing data on more than 14 million person-years. Combined with a high user-rate of progestogen-only contraception, this population-based study provides excellent generalisability and allows investigation of different subtypes of HC.

Compared with never users, we found no overall increased risk of developing breast cancer among current users of any combined HC, IRR 1.03 (0.91–1.16), whereas current users of progestogen-only methods had an increased risk of developing breast cancer, IRR 1.32 (1.20–1.45). Across all types of HC, the risk of developing breast cancer appeared to be highest the first five years of use (combined HC IRR 1.39 (1.14–1.69); progestogen-only methods IRR 1.74 (1.44–2.10)) and to disappear ten years after the women stopped using HC.

**Implications of all the available evidence**

Our data support an association between the use of progestogen-only methods and breast cancer development, but we only found a modest association for combined HC during the first five years of use. This may be explained by a more liberal prescription of progestogen-only methods to women with risk factors for breast cancer, such as smoking and obesity. Small risk estimates close to one are also likely to contribute to the inconsistent evidence on HC and breast cancer risk, and it remains unclear if the associations observed are casual.

As the risk of breast cancer is greatest among current users of HC whose prevalence of the disease is very low, the absolute risk of breast cancer among users of HC is minimal. However, given the extensive use of HC globally, more research is needed especially on progestogen-only methods, to optimize contraceptive guidelines and enable tailored contraceptive counselling to individual patients.

Alt-text: Unlabelled box

## Introduction

Hundreds of million women use hormonal contraception (HC) globally, and to many of these, the risk of developing breast cancer is a concern. The role of HC in breast cancer development has been debated for decades. Many studies have shown a small increased relative risk of breast cancer among current and recent users of combined oral contraception that disappears progressively within five to ten years after HC use is stopped.^1^, ^2^, ^3^, ^4^, ^5^, ^6^, ^7^, ^8^, ^9^ Other studies indicate no risk.^10^, ^11^, ^12^, ^13^ The discrepancies may be explained by the age and parity of the participants, prescription bias, study design (cohort versus case-control), and by definition of exposure (current use versus ever use).

Most prior studies are case-control studies, assessing the lifetime exposure to combined HC instead of current use, which is more relevant to women considering HC. In addition, many of the older studies are based on pills with higher ethinyl estradiol doses (≥50 mcg),^4^^,^^11^^,^^13^^,^^14^ formulations that are rarely used today. Updated research is needed as new HC formulations have arrived on the market. Some studies have suggested a lower risk of breast cancer among newer methods with lower oestrogen doses,^3^^,^^6^ although this association remains unclear.^1^^,^^10^^,^^15^ Evidence on breast cancer risk among users of non-oral HC and different formulations of HC is also scarce.^3^^,^^5^^,^^15^^,^^16^

In recent years, stemming from evidence on menopausal hormone therapy, an increased risk of breast cancer associated with methods containing progestogen has been detected.^2^^,^^17^^,^^18^ As the use of progestogen-only methods has been low in most parts of the world, former studies on HC and breast cancer have essentially only evaluated combined HC.^4^^,^^10^^,^^11^ For instance, in the Collaborative Group on Hormonal Factors in Breast Cancer study that reanalysed data from 54 epidemiological studies, only around 2% of participants had used progestogen-only methods.^1^ A few studies, mainly from the Nordic countries,^16^^,^^19^^,^^20^ have investigated the association between progestogen-only methods and breast cancer with inconsistent results. Sweden is unique in the sense that more women use progestogen-only methods than combined methods.^21^

Our aim was to study the risk of developing breast cancer among current users of HC, with a particular focus on progestogen-only methods.

## Methods

The regional ethical review board in Uppsala approved the study, DNR 2017/546.

### Study population

All Swedish women aged 15–34 at the study start on January 1^st^ 2005, or who turned 15 years during the study period, were followed until December 31^st^ 2017. The oldest women in the cohort were followed up to the age of 45. As the Swedish Prescribed Drug Register (SPDR, described below) started relatively recently, we only included women up to age 34 to avoid misclassification of older women as never-users. All Swedish residents have a unique identification number, which enables linking of information across several registers. We retrieved information from seven national registers: (1) The SPDR, (2) The National Patient Register with information on diagnoses classified according to the International Classification of Diseases and Related Health Problems version 10 (ICD-10), (3) The Swedish Medical Birth Register with information on virtually all deliveries and pregnancies from gestational week 22, (4) The Cause of Death Register with information on all dates and causes of all registered deaths, (5) The Total Population Register covering information on all Swedish residents registered as living in Sweden at any point since 1961, (6) The Multi-Generation Register containing information on individuals’ relation to biological or adoptive parents, and finally, (7) the Education Register.

Women were excluded if their mother had died from breast cancer or if they themselves had been diagnosed with breast cancer (ICD-10 codes D05, C50), gynaecological cancer (ICD-10 codes C51–C58), venous thromboembolism (VTE), stroke or cardiovascular disease (ICD-10 codes I10-I15, I20-I25, I26-I28, I42-I46, I49, I63, I74, I81-I82, O00-O07, O22, O87), or if they had emigrated. During the study, women were permanently censored if they died, emigrated or developed contraindications for combined HC use like gynaecological cancer, venous thromboembolism, coagulation defect, stroke, cardiovascular disease, systemic lupus erythematosus (ICD-10 code M32), or migraine with aura (ICD-10 code G43.1), whichever occurred first. Women were temporarily censored 12 months before giving birth and 6 months postpartum. Finally, we excluded the person-years for women aged 45 years or more as there were too few in this age group to make any viable analysis.

### Exposure

Information on redeemed prescriptions of HC between July 1st 2005 and December 31st 2017 was retrieved from the SPDR. The Anatomical Therapeutic Chemical (ATC) classification codes enabled drug identification. The SPDR covers all HC used by women in Sweden, as HC can only be prescribed by nurse-midwives or physicians. Prescription of HC for healthy women in Sweden is almost exclusively performed by nurse-midwives, and is free-of-charge up to age 20 or 25, depending on geographic area. Nurse-midwives must comply with the National Guidelines, which in turn are based on the Medical eligibility criteria for contraceptive use by the World Health Organization.^22^ According to the National Guidelines, nurse-midwives can only prescribe combined HC to women without risk factors for VTE or cardiovascular disease (for example body mass index (BMI) > 30.0 kg/m^2^ and smoking among women ≥35 years). Women with risk factors are either prescribed progestogen-only methods or are referred to a physician.

The defined daily dose (DDD) was used to estimate exposure time. Contraceptive patches and vaginal rings can be prescribed for three, six or twelve months. As the DDD is lacking in the SPDR for these methods, we assumed they were used for six months. Further, we assumed that the levonorgestrel-releasing intrauterine system (LNG-IUS) was used for four years, unless the woman was censored for pregnancy or because she started another HC method before the 4-year time period had ended. The LNG-IUS was analysed separately from the other progestogen-only methods.

Women were categorised as current users when they redeemed a prescription of HC during the study period. The current exposure time of dispensed prescriptions, except the LNG-IUS, were extended with 180 days as biological transformations may not be apparent until some months after starting use. Thus, the category ‘current users’ also include recent users. Women were categorised as previous users when the extension phase (i.e. DDD + 180 days) ended. We also performed additional analyses where ‘current users’ included DDD + 365 days to ensure sufficient time for biological transformation and detection of HC-induced cancers. Women who had not yet or who never redeemed a prescription of any HC during the study period were categorised as ‘never users’.

In the rare case where a woman retrieved two different oral HC the same day, both methods were censored until a new prescription was retrieved. If she retrieved an implant or LNG-IUS together with any type of oral contraception on the same day, she was presumed to have used the implant or the LNG-IUS. HC were categorised as shown in Table 1.

**Table 1:** Characteristics of the study population of women aged 15–34 years at study start.^a^

	No. of Person-Years	Ever users	Mean no of person-years^b^^,^^c^	Mean age^c^^,^^d^	Age at start^c^^,^^d^	Born in Sweden^c^^,^^e^	University^c^^,^^e^	Age at first birth^c^	Nulliparous women^c^	No of children^c^	BMI^c^^,^^f^	Smoking^c^^,^^f^
	Sum	%				%	%		%			Valid %
**All women**	14,330,806	*NA*	*NA*	*NA*	*NA*	86.8	41.9	27.3±4.6	58.6	2.0±0.9	24.9±4.7	8.5
Never used HC	6,320,610	31.1	4.0±3.8	25.7±8.1	24.2±7.6	84.3	47.4	27.7±4.5	50	2.0±0.9	24.8±4.8	8.1
Used HC >6 months previously	2,575,924	*NA*	3.2±2.7	29.5±7.1	28.3±6.8	89.7	51.3	27.0±4.4	44.6	1.9±0.9	24.8±4.7	8.8
Current or recent use of HC	5,434,272	68.8	4.8±3.1	26.0±7.4	25.5±7.2	91.6	48.6	26.8±4.3	52.6	1.9±0.8	24.8±4.7	8.6
**Current or recent use of all combined HC**^g^**^,^**^h^	2,874,157	47.8	3.6±2.8	23.5±5.9	23.1±5.9	91.9	49.6	26.9±4.4	64.7	1.8±0.8	24.2±4.1	7.2
COC with androgenic profile	1,529,144	32.5	2.9±2.5	21.9±5.3	21.5±5.3	92.5	45.6	26.2±4.3	70.2	1.7±0.8	24.3±4.2	7.8
Levonorgestrel COC	1,272,066	28.7	2.7±2.5	21.9±5.4	21.5±5.4	92.4	44.2	26.1±4.3	70.9	1.7±0.8	24.3±4.2	8.2
Norgestimat COC	2,58,735	7.5	2.1±2.1	21.8±4.6	21.4±4.6	93.6	50.4	26.4±4.0	69.5	1.7±0.7	24.0±4.1	6.4
COC with anti-androgenic profile	9,07,600	20.6	2.7±2.5	25.0±6.0	24.5±5.9	91.3	54.2	27.7±4.4	61	1.8±0.8	24.0±4.0	5.9
Drospirenon COC	7,00,516	17.5	2.4±2.3	24.5±5.9	24.0±5.8	91.8	53.3	27.3±4.3	64.9	1.8±0.8	24.0±4.0	5.8
Desogestrel COC	1,50,065	3.3	2.8±2.6	27.0±7.4	27.0±5.9	91.3	58.3	28.8±4.3	39.9	1.9±0.7	24.1±4.0	5.9
Estradiol COC	1,15,747	4.1	1.7±1.5	25.2±6.5	24.8±6.4	93.9	51.7	26.6±4.2	73	1.8±0.8	23.7±3.7	6.2
Patch or vaginal ring	3,36,202	9.6	2.1±2.0	24.9±5.6	24.4±5.5	91.9	52.5	26.5±4.3	58.3	1.8±0.8	24.2±4.0	8.1
**Current or recent use of progestogen-only products**^g^	1,755,803	36.6	2.9±2.7	26.7±7.4	26.0±7.0	91.5	44.0	26.6±4.4	47.7	2.0±0.9	25.3±5.1	10.7
Depot medroxyprogesterone acetate	1,27,211	2.4	3.3±3.3	31.6±7.3	30.1±6.8	88.4	24.5	25.2±4.4	37.3	2.2±1.0	26.2±5.6	24.1
Implant	4,71,267	9.9	2.9±2.3	23.6±6.2	22.9±6.0	90.2	33.7	24.9±4.3	62.6	1.9±0.9	25.6±5.1	13.6
Current or recent use of POP	1,171,636	30.2	2.4±2.5	27.1±7.3	26.4±7.0	92.2	47.8	26.9±4.4	45	2.0±0.9	25.2±5.1	9.1
NETA POP	90,476	3.1	1.8±2.2	29.3±6.8	28.4±6.5	91.9	54.6	27.2±4.3	32.7	2.1±0.8	24.7±4.7	7.6
Lynestrenol POP	76,970	2.5	1.8±2.3	29.7±6.7	28.8±6.3	91.7	54	27.5±4.3	28	2.1±0.9	24.7±4.8	7.1
Desogestrel POP	1,087,381	29.0	2.3±2.5	27.0±7.4	26.3±7.0	92.3	47.3	26.9±4.4	46.1	2.0±0.9	25.3±5.1	9.3
**LNG IUD**^g^	8,61,727	17.0	3.1±2.4	32.8±7.4	31.4±7.3	92.1	50.9	26.7±4.0	28.6	2.2±0.8	24.9±4.6	7.7

a All Swedish women aged 15–34 at the study start on January 1^st^ 2005 or who turned 15 years before the study stopped in December 31^st^ 2017 were included. Current use was defined as the exposure time of dispensed prescriptions (the defined daily dose) and extended with 180 days, thus the category also include recent users.

b Mean number of person-years that the same person used a certain method.

c Descriptive statistics were calculated as the average person-time with a certain characteristic, divided by the total amount of person-time during which hormonal contraception (HC) was used or not used. Plus-minus values are means ± standard deviation (SD). The percentages describe the percentages of person-time with a certain characteristic.

d Age at start of study or at start of using a HC method.

e Data on education were missing for 1,22,151 women (7.4%). Data on place of birth were missing for 24 women.

f Available for parous women only (37% of the study population). The body mass index (BMI) is calculated as the weight in kilograms divided by the square of the height in meters. BMI was measured for the most recent pregnancy when registering for antenatal care. Smoking was measured for the first pregnancy when registering for antenatal care.

g Combined oral contraception (COC), Progestogen-only pills (POP), Norethisterone (NETA), Intrauterine device (IUD).

h Including users of Dienogest COC and Cyproterone acetate COC, not analysed separately because of few cases.

### Outcome

The outcomes were any type of breast cancer (*in situ* or invasive) or death from breast cancer. These were defined as either the ICD-10 codes D05 (including both ductal and lobular cancer *in situ*) or C50, retrieved from the Patient Register, or the ICD-10 code C50 from the Swedish Cause of Death Register. Additional analyses were performed with invasive breast cancer-only as outcome.

### Covariates

Information on age and place of birth was collected from the Total Population Register, and information on level of education was retrieved from the Swedish Education Register. We collected information on parity, age at first birth, smoking, and BMI from the Swedish Medical Birth Register (available in 37% of the study population). Women with no information in the Swedish Medical Birth Register were assumed to be nulliparous. Information on smoking was collected when registering for antenatal care for the first pregnancy, and information on BMI was collected from the most recent pregnancy. Finally, the National Patient Register provided information on any infertility diagnosis (ICD-10 N97), polycystic ovarian syndrome (ICD-10 E282 and L680) and endometriosis (ICD-10 N80), and the SPDR provided information on ovulation stimulating treatment (ATC G03G).

### Statistical analysis

We calculated the crude and adjusted incidence rate ratios (IRR) of breast cancer among different user groups of HC using Poisson regression models with incidence of breast cancer or breast cancer *in situ* as event. Age was used as a time-varying variable, with 5-year age bands. The reference group was women who had never used HC. The individual woman could contribute both as never, current and previous user of HC. A two-sided p-value of less than 0.05 was considered significant in all analyses and IRR are presented with 95% confidence intervals (CI).

We performed the adjustments for covariates in two models. In Model 1, we adjusted for age, level of education (elementary school, upper secondary school, post-secondary education or university, or missing), place of birth (Sweden, Europe, outside Europe, or missing), age at first birth (<25, 25<0, ≥30 years, or missing/nulliparous), number of children (0, 1, 2, 3, ≥4 children) and any diagnosis that suggested HC was used for medical purposes (infertility, polycystic ovary syndrome, and endometriosis) or ovulation stimulating treatment. In Model 2, we added information on BMI and smoking; these data were assessed as complete-cases in the analyses and included parous women only. All variables were treated as categorical except BMI and age that were entered as continuous variables.

We investigated the effect of duration of use and time since last use by presenting the age-adjusted IRR using Poisson regression. We studied the (1) duration of current episode of use (2) the cumulative duration of use, and (3) time since last use, all compared with all never users. Women were grouped into use of <5 years, 5<10 years and ≥10 years. Women were allowed to contribute in all three groups.

All analyses were performed using R Statistics version 4.1.0 except the descriptive statistics that were performed in IBM SPSS version 28.0. All IRR in the results section are adjusted according to Model 1 if not otherwise specified.

### Role of funding source

The funding sources were not involved in the conduct of the study.

## Results

**Figure 1:**
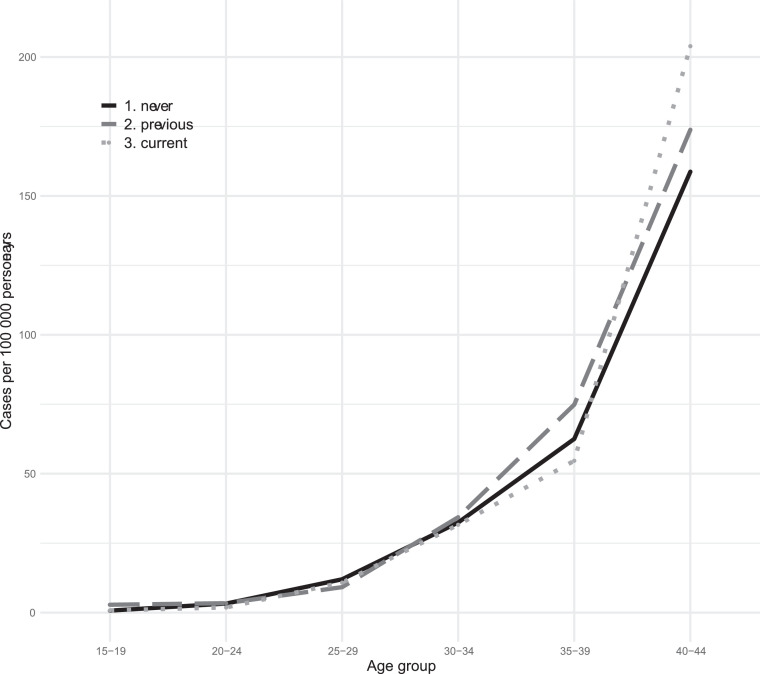
Breast cancer incidence among different age groups of Swedish women who are current, previous or never users of combined hormonal contraception.

**Figure 2:**
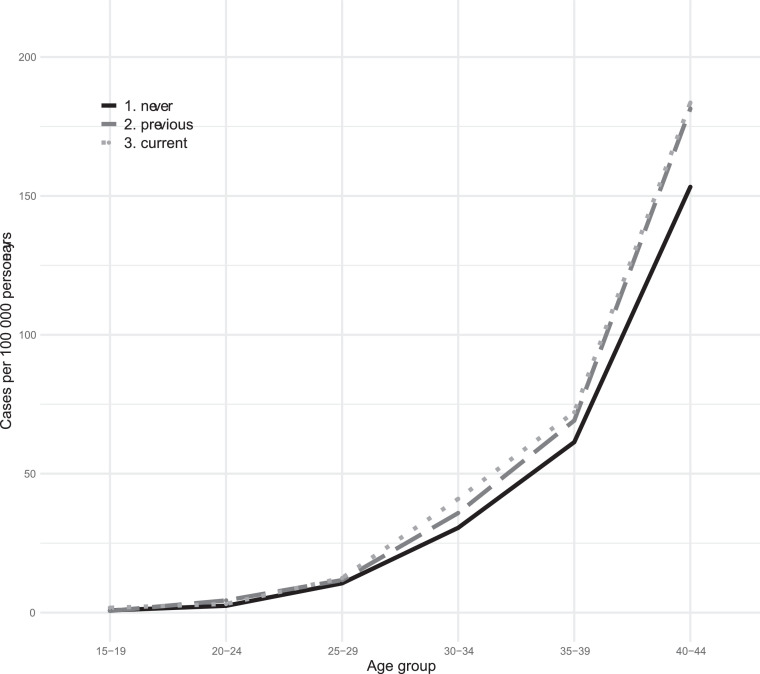
Breast cancer incidence among different age groups of Swedish women who are current, previous or never users of progestogen-only methods.

This cohort included 1,652,364 women followed from 2005 to 2017 with a mean follow-up time of 8.7±3.9 years and a total of 14,330,806 person-years. During this time period, 3842 events of breast cancer were recorded, of which 495 cases were breast cancers *in situ* and 3346 were breast cancers. Of the 495 cases with breast cancer *in situ*, 261 later developed breast cancer. One death from breast cancer, not previously diagnosed, was recorded. This generated a breast cancer incidence of 26.8 per 100,000 women-years in this population. Overall, 3.2% of the breast cancers occurred among women aged 15–25, 27.3% among women aged 25–35 and 69.5% among women aged ≥35 years, Figure 1, Figure 2.

Women were between 15.0 to 45.0 years of age and the mean age was 25.7±7.4 years. The distribution of person-years in the age groups < 20, 20–29.9, 30–39.9 and > 40 were 23.4 %, 43.9 % 27.5 % and 5.2%, respectively. Almost half of all women had used combined HC (47.8%) and these women were generally younger, had a lower BMI, and were more often nulliparous compared with the remaining women. Around 36.6% of all women had used progestogen-only methods. These women were more often parous, had a higher BMI, and were more often smokers compared with the remaining women. The study population is described in Table 1.

### Combined hormonal contraception

Current users of any combined HC method had no increased risk of developing breast cancer or breast cancer *in situ* (IRR 1.03 (0.91–1.16) (Table 2, model 1)). None of the individual combined HC preparations conferred any increased risk of developing breast cancer (Table 2). Analyses in women with invasive breast cancer only, and analyses with increased time-lag for current use set to 12 months yielded similar findings (Supplementary Tables 1 and 2).

**Table 2:** Incidence rate ratios (IRR, referred to as relative risk) of breast cancer and breast cancer in situ among women aged 15–34 years at study start in relation to different types of hormonal contraception (HC).

	No of breast cancer events	No of person-years	IRR	95% CI	*P*	IRR	95% CI	*P*	Absolute risk (%)^c^	No of breast cancer events	No of person-years	IRR	95% CI	*P*
	Crude					Model 1 a				Model 2 b				
Never used HC	1419	6,322,450	1.00			**1.00**	reference		22.4	851	2,459,880	1.00	reference	
Used HC >6 months previously	1068	2,576,957	1.19	1.10–1.29	<0.01	**1.26**	1.17–1.37	<0.01	41.4	700	1,283,280	1.19	1.08–1.32	<0.01
Current or recent use of HC d	1355	5,436,492	1.20	1.12–1.30	<0.01	**1.34**	1.24–1.44	<0.01	24.9	874	2,090,847	1.22	1.11–1.34	<0.01
**Current or recent use of any combined HC**^d^^,^^e^	314	2,875,409	0.98	0.86–1.10	0.69	1.03	0.91–1.16	0.67	10.9	141	8,22,971	0.82	0.69–0.97	0.02
COC with androgenic profile^d^	104	1,529,794	0.89	0.72–1.07	0.23	0.92	0.75–1.12	0.41	6.8	44	3,84,819	0.75	0.55–1.01	0.06
Levonorgestrel COC d	90	1,272,596	0.88	0.71–1.09	0.25	0.92	0.74–1.14	0.43	7.1	37	3,09,965	0.72	0.52–1.00	0.05
Norgestimat COC d	15	2,58,858	0.90	0.52–1.45	0.70	0.94	0.56–1.56	0.80	5.8	8	76,396	0.97	0.48–1.94	0.92
COC with anti-androgenic profile^d^	138	9,08,024	0.97	0.81–1.15	0.73	1.02	0.86–1.21	0.84	15.2	64	3,08,109	0.82	0.64–1.05	0.12
Drospirenon COC d	99	7,00,846	1.03	0.84–1.25	0.79	1.08	0.89–1.33	0.44	14.1	47	2,12,267	0.91	0.68–1.22	0.54
Desogestrel COC d	34	1,50,135	0.91	0.64–1.25	0.58	0.92	0.66–1.30	0.65	22.6	17	78,761	0.76	0.47–1.23	0.27
Estradiol COC d	25	1,15,802	1.19	0.78–1.72	0.39	1.26	0.85–1.87	0.24	21.6	10	24,906	0.87	0.47–1.62	0.66
Patch or vaginal ring	50	3,36,367	1.05	0.79–1.38	0.72	1.12	0.84–1.48	0.44	14.9	25	1,18,543	0.93	0.63–1.38	0.72
**Current or recent use of progestogen-only methods**	523	1,756,551	1.23	1.11–1.35	*p*<0.01	**1.32**	1.20–1.45	<0.01	29.8	324	7,43,402	1.26	1.12–1.42	<0.01
Depot injection	50	1,27,259	0.79	0.59–1.03	0.09	**0.74**	0.56–0.97	0.03	39.3	27	64,031	0.81	0.55–1.19	0.28
Implant	78	4,71,485	1.22	0.97–1.51	0.08	**1.31**	1.05–1.64	0.02	16.5	47	1,53,514	1.28	0.96–1.71	0.10
Any POP d	398	1,172,132	1.28	1.15–1.42	<0.01	**1.40**	1.26–1.56	<0.01	34.0	252	5,38,893	1.28	1.12–1.47	<0.01
NETA POP d	44	90,515	1.37	1.00–1.82	0.04	**1.47**	1.09–1.98	0.01	48.6	32	54,041	1.43	1.01–2.03	0.04
Lynestrenol POP d	28	76,003	0.98	0.66–1.39	0.92	1.06	0.73–1.53	0.77	36.8	22	48,055	1.11	0.73–1.69	0.63
Desogestrel POP d	356	1,087,842	1.25	1.12–1.40	<0.01	**1.37**	1.22–1.53	<0.01	32.7	222	4,90,434	1.24	1.08–1.43	<0.01
**Levonorgestrel IUD**^d^	543	8,62,041	1.10	1.01–1.21	0.04	**1.21**	1.01–1.33	<0.01	63.0	433	5,76,119	1.20	1.08–1.34	<0.01

a Adjusted for age at start of each exposure, level of education, place of birth, age at first full-term pregnancy, number of children, having received ovulation stimulating treatment and any diagnosis of infertility, polycystic ovarian syndrome, or endometriosis.

b Adjusted for the same covariates as Model 1, including for body-mass index (BMI) and smoking (available for parous women only; 37% of the study population).

c Absolute risks were calculated as the number of breast cancer events divided by the number of person-years, multiplied with 100,000.

d Hormonal contraception (HC), Combined oral contraception (COC), Progestogen-only pills (POP), Norethisterone (NETA), Intrauterine device (IUD).

e Including users of Dienogest COC and Cyproterone acetate COC, not analysed separately because of few cases.

However, a modest risk of breast cancer with combined HC was noted the first five years of use (IRR 1.39 (1.14–1.69)), which disappeared after five years of use (Table 3A). The risk of breast cancer again became apparent the first ten years since most recent use (< 5 years; IRR 1.66 (1.45–1.90) and 5–10 years after stopped use; IRR 1.22 (1.08–1.38)), but was no longer significant ten years after stopped use (Table 3A).

**Table 3A:** Incidence rate ratio (IRR, referred to as relative risk) of breast cancer among women aged 15-34 years at study start, according to duration of current combined hormonal contraception (HC) use (1), time since most recent use (2), and cumulative duration (3).

	No of breast cancer events	No of person-years	Incidence rate	Age-adjustedincidence rate/100,000 person-years	IRR	95% CI	*p*	No of breast cancer events	No of person-years	IRR	95% CI	*p*
	Crude estimates			Adjusted estimates– Model 1^a^				Model 2^b^				
**Never used HC**	1419	6,322,450	22.4	20.4	1.00	Reference	*NA*	851	2,459,880	1.00	Reference	*NA*
**Duration of combined HC use**^b^												
0 < 5 years	114	1,345,829	8.5	25.9	**1.39**	1.14–1.69	<0.01	77	4,47,054	**1.32**	1.03–1.68	0.03
5 < 10 years	132	1,213,245	10.9	20.8	1.06	0.88–1.13	0.53	45	3,35,251	**0.66**	0.49–0.90	<0.01
≥ 10 years	68	3,18,304	21.4	16.8	**0.78**	0.61–1.00	0.05	19	41,230	0.70	0.44–1.10	0.05
**Time since most recent use**												
0 < 5 years	261	7,84,184	33.3	39.6	**1.66**	1.45–1.90	<0.01	221	5,00,231	**1.72**	1.47–2.00	<0.01
5 < 10 years	293	1,089,677	26.9	39.5	**1.32**	1.16–1.50	<0.01	172	4,17,879	1.12	0.95–1.33	0.16
≥ 10 years	238	6,92,160	34.4	18.2	**0.85**	0.74–0.98	0.02	104	1,77,845	**0.77**	0.63–0.96	0.02
**Cumulative duration of combined HC use**^c^												
0 < 5 years	68	1,237,573	5.5	21.3	1.30	1.00–1.67	0.04	35	3,47,758	1.03	0.73–1.46	0.87
5 < 10 years	171	1,290,061	13.3	23.9	1.23	1.04–1.45	0.01	80	4,05,607	0.99	0.78–1.25	0.95
≥ 10 years	75	3,49,744	21.4	15.8	**0.75**	0.59–0.94	0.01	26	70,170	**0.67**	0.45–0.99	0.04

a Adjusted for age at start of each exposure, level of education, place of birth, age at first pregnancy registered for antenatal care, number of children, having received ovulation stimulating treatment, and any diagnosis of infertility, polycystic ovarian syndrome, or endometriosis.

b Adjusted for the same covariates as Model 1, including for body-mass index (BMI) and smoking (available for parous women only; 37% of the study population).

c Cumulative time per person during the study period.

### Progestogen-only hormonal contraception

Current users of progestogen-only methods had an increased risk of developing breast cancer or breast cancer *in situ* in comparison with never users of any HC, IRR 1.32 (1.20–1.45) (Table 2, model 1). The risk was increased across all progestogen-only methods, except users of the depot medroxyprogesterone acetate injectable who had a slightly lower risk, IRR 0.75 (0.56–0.97). The greatest risk of developing breast cancer was observed among users of the progestogen-only pills, IRR 1.40 (1.26–1.56). When adjusting for available information on BMI and smoking, this risk was somewhat attenuated, IRR 1.28 (1.12–1.47) among parous women (Table 2, model 2).

Analyses in women with invasive breast cancer only, and analyses where we increased the time-lag for current use to 12 months yielded similar findings (Supplementary Tables 1 and 2).

The risk of breast cancer with progestogen-only methods was highest the first ten years of use (0 – 5 years; IRR 1.74 (1.44–2.10) and 5–10 years; IRR 1.26 (1.06–1.49)) and diminished after ten years of use (Table 3B). This increased risk remained apparent the first ten years after stopped use. Ten years after stopped use, we could no longer detect any increased risk for breast cancer with the progestogen-only methods (Table 3B).

**Table 3B:** Incidence rate ratio (IRR, referred to as relative risk) of breast cancer among women aged 15-34 years at study start, according to duration of progestogen-only methods use (1), time since most recent use (2), and cumulative duration of progestogen-only methods (3).

	No of breast cancer events	No of person-years	Incidence rate	Age-adjusted incidence rateNo of events per 100,000 person-years	IRR	95% CI	*p*	No of breast cancer events	No of person-years	IRR	95% CI	*p*
	Crude estimates			Adjusted estimates - Model 1^a^				Model 2^b^				
**Never used HC**	1419	6,322,450	22.4	20.4	1.00	Reference	*NA*	851	2,459,880	1.00	Reference	*NA*
**Duration of progestogen-only methods use**^b^												
0 < 5 years	157	5,45,405	28.8	39.6	**1.74**	1.48–2.05	<0.01	120	3,06,196	**1.74**	1.44–2.10	<0.01
5 < 10 years	143	4,50,423	31.7	27.2	**1.26**	1.06–1.49	<0.01	90	1,83,069	1.21	0.97–1.49	0.09
≥ 10 years	98	1,77,107	55.3	24.8	0.91	0.74–1.11	0.35	42	49,997	0.78	0.57–1.06	0.11
**Time since most recent use**												
0 < 5 years	300	6,50,200	46.1	40.0	**1.93**	1.71–2.18	<0.01	265	4,47,815	**2.14**	1.87–2.46	<0.01
5 < 10 years	317	8,66,461	36.6	25.5	**1.25**	1.00–1.26	0.05	214	4,28,979	1.02	0.88–1.17	0.83
≥ 10 years	191	4,72,809	40.4	18.0	**0.73**	0.63–0.84	<0.01	105	1,54,976	**0.70**	0.57–0.86	<0.01
**Cumulative duration of progestogen-only methods**^b^												
0 < 5 years	93	4,14,040	22.5	47.7	**2.14**	1.74–2.64	<0.01	64	1,85,586	**2.08**	1.61–2.68	<0.01
5 < 10 years	191	5,45,020	35.0	29.5	**1.42**	1.22–1.64	<0.01	130	2,70,076	**1.43**	1.19–1.71	<0.01
≥ 10 years	114	2,13,875	53.3	23.1	0.85	0.71–1.03	0.10	58	83,600	0.76	0.59–1.00	0.05

a Adjusted for age at start of each exposure, level of education, place of birth, age at first pregnancy registered for antenatal care, number of children, having received ovulation stimulating treatment, and any diagnosis of infertility, polycystic ovarian syndrome, or endometriosis.

b Adjusted for the same covariates as Model 1, including for body-mass index (BMI) and smoking (available for parous women only; 37% of the study population). ^c^Cumulative time per person during the study period.

To rule out the possibility that the elevated risk of developing breast cancer among users of progestogen-only contraception was due to recent prior use of combined HC, we compared women who had exclusively used progestogen-only methods with never users. The risk of developing breast cancer in exclusive progestogen-only contraceptive users remained elevated in this analysis, IRR 1.29 (1.07–1.56).

We also evaluated risk of breast cancer in foreign-born women, nulliparous women and women under the age of 20. Due to few cases, we performed the analyses with any hormonal contraception as exposure. We found an increased risk among women under the age of 20 years, IRR 4.03 (1.33–12.21), but the absolute risk in these women was only 0.8/1,00,000 person-years (Supplementary Table 3).

## Discussion

### Main findings and interpretation

Swedish women who use progestogen-only methods have a marginally increased relative risk of developing breast cancer or breast cancer *in situ* compared with women who have never used HC. We could not detect any overall increased risk among users of combined HC, except a slightly increased risk during the first five years of use. The risk of breast cancer appeared to be highest during the first ten years of progestogen-only methods use. Across all HC, the risk of developing breast cancer disappeared within ten years after stopped use. Although we demonstrate an increased relative risk of developing breast cancer, the absolute risk of acquiring breast cancer from combined HC or progestogen-only method use was small in this population of women 15–34 years of age at study start.

### Results on combined contraception

We could not identify any overall increased risk of developing breast cancer among current users of combined HC or in any of the different combined HC formulations. This contrasts to the findings by the Collaborative Group,^1^ and to a Danish study that identified an increased risk among several of the combined oral contraceptives.^16^ The reason for this discrepancy may be explained by several factors. Firstly, many high quality studies have not identified any increased relative risk,^10^, ^11^, ^12^, ^13^ and those that have, present small estimates with confidence intervals close to one.^1^, ^2^, ^3^, ^4^, ^5^, ^6^, ^7^, ^8^, ^9^ Secondly, the prescription of HC for contraception in Sweden is almost exclusively performed by nurse-midwives who are only allowed to prescribe combined HC to women who are not obese or smoking (if the woman is aged 35 years or more). Women in this cohort who used combined HC were younger, had a higher educational level, lower BMI, and were smoking to a lesser extent. Women with risk factors for breast cancer, such as obesity or smoking, are either prescribed progestogen-only methods or are referred to a physician, creating a selection bias towards healthier combined HC users.

### Results on progestogen-only methods

We identified an increased risk of developing breast cancer among current users of progestogen-only methods as well as among exclusive users of progestogen-only methods. The increased risk was consistent across all formulations for which we had adequate statistical power. We could not detect any major differences between the different formulations, except for the depot medroxyprogesterone acetate (DMPA) injectable.

As progestogen-only methods have previously not been widely used globally, there are only a few studies to compare with. In the Norwegian Women and Cancer (NOWAC) Cohort Study, users of progestogen-only methods had similar relative risk of developing breast cancer as found in our study.^19^ Among users who exclusively had used progestogen-only methods, the risk estimate was even higher.^19^ However, use in the NOWAC study was based on self-report and only low-dose formulations were registered. In a Danish study, an increased relative risk of developing breast cancer could be detected only in a few progestogen-only methods,^16^ but the number of events in each category were small and may have been underpowered to detect any associations.

Current users of DMPA had a decreased risk of developing breast cancer compared with never users of HC. Evidence from former studies also points towards no increased risk for breast cancer in this group, although findings are not entirely convincing.^1^^,^^23^^,^^24^ As very few women in Sweden use DMPA today, this finding should be interpreted with great caution.

Current use of the LNG-IUS was associated with a small increased risk of developing breast cancer compared with never users. This association was also prevalent in a Danish study as well as in a meta-analysis, in which the odds ratio was 1.12 for premenopausal women.^16^^,^^25^ In contrast, the NOWAC study identified no increased risk of breast cancer among users of the LNG-IUS.^26^ We may have overestimated the hormonal exposure of the intrauterine device as we assumed all women had used it for four years. Also, we cannot determine if women used the LNG-IUS as part of menopausal hormonal therapy.

On the one hand it is not surprising that progestogen-only methods increase the risk of developing breast cancer, as women using oral progestogen and implants maintain their endogenous production of oestrogen (although at lower levels compared with ovulatory menstrual cycles) and as progestogen stimulate cell proliferation. On the other hand, users of progestogen-only methods in our cohort constitute a selected group, as discussed above. The latter is further noted when we adjust for BMI and smoking, by which risk estimates for breast cancer became somewhat attenuated. As BMI was only available in 37% of the study population, we cannot rule out that this finding is affected by residual confounding, including not only BMI, but also health literacy, physical activity, dietary patterns, alcohol consumption, age at menarche, and breastfeeding.

The effect by which hormonal contraceptives are thought to influence breast cancer risk could be due to direct stimulatory action on breast tissue, for instance, via cross regulatory signalling networks between the estrogen receptor (ER) α and BRCA1,^27^ or indirectly via delayed childbirth and lower parity. Recently a meta-analysis pointed out that HC-induced risk was tied to breast cancer receptor status, where increased risks were noted for women with ER negative and triplet-negative breast cancer.^28^ In contrast, a reduced risk from HC use was noted in ER positive cancers.^28^

### Time effects of combined HC use and POP use

We found a marginally increased risk of developing breast cancer in the first five years of combined HC use, and during the first ten years of POP use, which in both contraceptive groups diminished with longer duration of use. Studies on a time- and dose-dependent effect of combined HC on breast cancer development have given inconsistent results; some have found a moderate effect that appears at various time points,^5^^,^^16^^,^^19^ while two systematic reviews suggest no or no certain effect.^1^^,^^10^ As our risk estimates were small, had overlapping confidence intervals and come from observational data, they cannot be relied on for demonstrating the casual nature of a time- and dose-related response.^29^ In addition, the group of women using combined HC for more than ten years was small because of the short follow-up time.

In line with former studies,^1^^,^^16^ we found that previous users of combined HC and POP had a slightly increased risk of breast cancer that persisted up to ten years after stopped use. The different estimates observed in ours versus prior studies may be explained by the diverse distribution of HC methods in the different cohorts. Our finding that the breast cancer risk disappears ten years after stopped use is biologically plausible and contributes to the body of evidence suggesting that former use of HC does not increase the risk of breast cancer later in life.^1^^,^^4^^,^^9^^,^^10^^,^^16^

### Strengths and limitations

This large, nationwide prospective cohort study included almost 1.7 million women who were followed for thirteen years, which provided us data on more than fourteen million person-years. More than 2.6 million person-years on progestogen-only methods were included, which is more than in any prior study. The use of national registers is a strength as they cover all Swedish women, all cases of diagnosed breast cancer and all redeemed prescriptions of HC in Sweden. In comparison to our study, most former evidence on the association between HC and breast cancer comes from case-control studies, which are susceptible to recall bias and have limited generalisability.

We included reliable information from the national registers on several potential confounders, including variables that are strongly associated with breast cancer risk.^30^ These included e.g. age, age at first birth, and parity. Data on smoking and BMI were incomplete as this information was available only in parous women. However, information on important confounders or risk factors such as family history of breast cancer, BRCA mutations, benign breast disease, mammographic density, alcohol consumption or physical activity are missing. A few heterogeneous findings are observed in this report, but as it contains some 100 IRR and their respective confidence intervals, at least part of this variation in risk between subgroups is likely due to chance.

The incidence of breast cancer was assessed using The National Patient Register. This register only covers diagnoses reported by a clinician and not from the pathology laboratory. The major advantage of the Cancer Register is the quality check with the pathology register, which means it only includes incident cases. With our young study population, however, most breast cancer are bound to be incident, i.e. we find it unlikely that the diagnoses merely reflect history of breast cancer. Comparing our data with data from the National Cancer Register with 99% coverage of breast cancer diagnoses,^31^ we had the same proportions of *in situ* versus invasive cases (12.8% vs 12.8%).^32^ Further, as we have no reason to believe that there is any differential reporting of breast cancer among HC users and never users, we do not believe this have impacted our findings. However, important information from the Cancer Register such as receptor status, stage and pathology would have given a more complete picture of the associations.^28^ Final limitations of this study is that we followed the women for a relatively short period of time, and we only included women up to 34 years of age at the start of the study. As progestogen-only methods are the most common contraceptives in women 35–50 years of age, and as the absolute risk of breast cancer increases at this age, we may have underestimated the risk of using progestogen-only methods. Further, as women 35–50 years of age were not included, we may have underestimated the effect of longer duration of use.

## Conclusions

Swedish women in our cohort of women, 15–34 years of age, who currently use progestogen-only methods have a small increased relative risk of developing breast cancer or breast cancer *in situ* compared with women who have never used hormonal contraceptives. In contrast, we could not detect any overall increased risk among current users of combined HC. However, the risk with combined HC and progestogen-only methods appears to be greatest during the first five and ten years of use, respectively. Across all HC, the risk of breast cancer persisted up to ten years after stopped use. As the risk of breast cancer is greatest among current users of HC whose prevalence of the disease is very low, the absolute risk of breast cancer among users of HC is minimal. However, given the extensive use of HC globally, more research is needed especially on progestogen-only methods, to optimize contraceptive guidelines and enable tailored contraceptive counselling to individual patients. At the same time, the many health benefits associated with HC must also be taken into account in contraceptive counselling.

## Contributors

Jenny Niemeyer Hultstrand was involved in the following study activities: conceptualisation, data curation, formal analysis, investigation, interpretation of results, methodology, project administration, software, validation, interpretation, visualization, writing manuscript.

Kristina Gemzell-Danielsson was involved in the following study activities: conceptualisation, validation, interpretation of results and writing (review and editing).

Helena Kopp Kallner was involved in the following study activities: conceptualisation, validation, interpretation of results and writing (review and editing).

Henrik Lindman was involved in the following study activities: conceptualisation, validation, interpretation of results and writing (review and editing).

Per Wikman was involved in the following study activities: data curation, formal analysis, investigation, software, validation and visualization.

Inger Sundström Poromaa was involved in the following study activities: conceptualisation, data curation, formal analysis, funding acquisition, investigation, methodology, project administration, resources, supervision, validation, interpretation of results and writing (review and editing).

## Data sharing statement

No individual participant data can be made available or shared. Study protocol and data dictionaries are available upon contact with the corresponding author.

## Declaration of interests

Jenny Niemeyer Hultstrand has received funding from the Family Planning Fund, Uppsala University, and travel grants from the Swedish Society of Medicine.

Kristina Gemzell-Danielsson has acted as consultant for Bayer, Gedeon Richter, Exeltis, Natural Cycles, Daisy, Medincell, Cirqle, ObsEva, Mithra, Vifor. She has received lecture fees from Bayer, Gedeon Richter, Exeltis, Nordic Pharma, Natural Cycles, Mithra, and Organon.

Helena Kopp Kallner has received lecture fees from: Actavis, Bayer, Gedeon Richter Exeltis, Nordic Pharma, Natural Cycles, Mithra, Teva, Merck, Ferring, Consilient Health, and acted as consultant for Bayer, Evolan, Gedeon Richter, Exeltis, Merck, Teva, TV4, Natural Cycles, Pharmiva, Dynamic Code, Ellen, Estercare, Leia and Essity.

Henrik Lindman has received lecture fees from Lilly, AstraZeneca, Novartis, and Daiichi Sankyo.

Per Wikman reports no conflicts of interest.

Inger Sundström Poromaa has served occasionally on advisory boards or acted as invited speaker at scientific meetings for Bayer Health Care, Gedeon Richter, Peptonics, Shire/Takeda, and Sandoz. ISP is supported by grants from the Swedish Research Council 2020-01801 and the Swedish Cancer Society.

## Appendix Supplementary materials

Image, application 1

Image, application 2

Image, application 3

Image, application 4
