# Supplementary material for: Hormonal contraception and risk of breast cancer and breast cancer in situ among Swedish women 15–34 years of age: A nationwide register-based study
Source: Lancet Reg Health Eur. 2022 Jul 29;21:100470. doi: 10.1016/j.lanepe.2022.100470 (PMC9340531; doi:10.1016/j.lanepe.2022.100470)
Supplement: Supplementary file 1 [file mmc1.docx]

**Supplementary Table 1. Incidence rate ratios (IRR, referred to as relative risk) of breast cancer-only among women aged 15-34 years at study start using different types of hormonal contraception (HC).**

|  | **No of breast cancer events** | **No of person-years** | **IRR** | **95% CI** | **P** | **IRR** | **95% CI** | **P** | **Absolute risk per 100,000^c^** | **No of breast cancer events** | |  | | **IRR** | **95% CI** | **P** |
| --- | --- | --- | --- | --- | --- | --- | --- | --- | --- | --- | --- | --- | --- | --- | --- | --- |
| **Crude** | | | | | | **Model 1^a^** | | | | |  | | **Model 2^b^** | | | |
| Never used HC | 1,325 | 6,327,215 | 1·00 |  |  | **1·00** | reference |  | 20·9 | 828 | |  | | 1·00 | reference |  |
| Used any HC >6 months previously | 1,007 | 2,578,980 | 1·95 | 1·79-2·11 | <0·01 | **1·19** | 1·08-1·33 | <0·01 | 39·0 | 695 | |  | | 1·17 | 1·04-1·30 | <0·01 |
| Current or recent use of any HC | 1,276 | 5,440,415 | 1·21 | 1·13-1·32 | <0·01 | **1·25** | 1·13-1·38 | <0·01 | 23·5 | 865 | |  | | 1·21 | 1·08-1·34 | <0·01 |
|  |  |  |  |  |  |  |  |  |  |  | |  | |  |  |  |
| Current or recent use of any combined HC | 301 | 2,961,792 | 0·79 | 0·79-0·85 | <0·01 | 0·95 | 0·80-1·13 | 0·57 | 10·9 | 137 | |  | | 0·91 | 0·76-1·09 | 0·31 |
|  |  |  |  |  |  |  |  |  |  |  | |  | |  |  |  |
| Current or recent use of progestogen-only methods | 492 | 1,757,814 | 1·46 | 1·32-1·46 | p<0·01 | **1·22** | 1·08-1·38 | <0·01 | 29·8 | 246 | |  | | 1·25 | 1·09-1·43 | <0·01 |

^a^ Adjusted for age at start of each exposure, level of education, place of birth, age at first full-term pregnancy, number of children, having received ovulation stimulating treatment and any diagnosis of infertility, polycystic ovarian syndrome, or endometriosis.

^b^ Adjusted for the same covariates as Model 1, including for body-mass index (BMI) and smoking (available for parous women only; 37% of the study population).

^c^ Absolute risks were calculated as the number of breast cancer events divided by the number of person-years, multiplied with 100,000.
