# Supplementary material for: Hormonal contraception and risk of breast cancer and breast cancer in situ among Swedish women 15–34 years of age: A nationwide register-based study
Source: Lancet Reg Health Eur. 2022 Jul 29;21:100470. doi: 10.1016/j.lanepe.2022.100470 (PMC9340531; doi:10.1016/j.lanepe.2022.100470)
Supplement: Supplementary file 2 [file mmc2.docx]

**Supplementary Table 2. Incidence rate ratios (IRR) of breast cancer and breast cancer in situ among women aged 15-34 years at study start using different types of hormonal contraception (HC), extending recent use up to 12 months after prescription ended.**

|  | **No of breast cancer events** | **No of person-years** | **IRR** | **95% CI** | **P** | **IRR** | **95% CI** | **P** | **Absolute risk per 100,000^c^** | **No of breast cancer events** | | **IRR** | **95% CI** | **P** |
| --- | --- | --- | --- | --- | --- | --- | --- | --- | --- | --- | --- | --- | --- | --- |
| **Crude** | | | | | | **Model 1^a^** | | | | | **Model 2^b^** | | | |
| Never used HC | 1,419 | 6,326,784 | 1·00 |  |  | **1·00** | reference |  | 22·0 | 851 | | 1·00 | reference |  |
| Used HC >12 months previously | 979 | 2,158,905 | 2·19 | 2·02-2·38 | <0·01 | **1·20** | 1·09-1·33 | <0·01 | 45·3 | 654 | | 1·18 | 1·06-1·32 | <0·01 |
| Current or recent use of any HC^d^ | 1,444 | 5,860,028 | 1·16 | 1·08-1·25 | <0·01 | **1·23** | 1·11-1·35 | <0·01 | 24·6 | 920 | | 1·21 | 1·09-1·33 | <0·01 |
|  |  |  |  |  |  |  |  |  |  |  | |  |  |  |
| Current or recent use of any combined HC^d^ | 358 | 3,144,849 | 0·78 | 0·73-0·82 | <0·01 | 0·92 | 0·79-1·09 | 0·29 | 11·4 | 158 | | 0·85 | 0·72-1·02 | 0·07 |
|  |  |  |  |  |  |  |  |  |  |  | |  |  |  |
| Current or recent use of progestogen-only methods^d^ | 523 | 1,756,551 | 1·23 | 1·11-1·35 | p<0·01 | **1·32** | 1·20-1·45 | <0·01 | 29·8 | 361 | | 1·26 | 1·12-1·42 | <0·01 |

^a^ Adjusted for age at start of each exposure, level of education, place of birth, age at first full-term pregnancy, number of children, having received ovulation stimulating treatment and any diagnosis of infertility, polycystic ovarian syndrome, or endometriosis.

^b^ Adjusted for the same covariates as Model 1, including for body-mass index (BMI) and smoking (available for parous women only; 37% of the study population).

^c^ Absolute risks were calculated as the number of breast cancer events divided by the number of person-years, multiplied with 100,000.

^d^ Recent use extended to 12 months of hormonal contraception
