# Supplementary material for: Hormonal contraception and risk of breast cancer and breast cancer in situ among Swedish women 15–34 years of age: A nationwide register-based study
Source: Lancet Reg Health Eur. 2022 Jul 29;21:100470. doi: 10.1016/j.lanepe.2022.100470 (PMC9340531; doi:10.1016/j.lanepe.2022.100470)
Supplement: Supplementary file 3 [file mmc3.docx]

**Supplementary table 3. Incidence rate ratios (IRR) of breast cancer and breast cancer in situ among women aged 15-34 years at study start using any hormonal contraception (HC). Subgroup analyses in (1) foreign-, and Swedish-born women, (2) nulliparous women and (3) women < 35 years of age and (4) women < 20 years of age.**

|  | **Variable** | **No of person-years** | **Events** | **Crude IRR** | **p-value** | **Model 1^a^**  **Adjusted IRR** | **p-value** | **Model 2^b^**  **Adjusted IRR** | **p-value** |
| --- | --- | --- | --- | --- | --- | --- | --- | --- | --- |
| Foreign-born women | Never used HC |  |  | 1·00 (reference) |  | 1,00 (reference) |  | 1,00 (reference) |  |
|  | Current or recent use of HC | 393,079 | 153 | 1·24 (1·02-1·50) | 0·029 | 1·23 (1·01-1·49) | 0·039 | 1·15 (0·89-1·47) | 0·286 |
|  | Used HC >6 months previously | 303,298 | 171 | 1·76 (1·47-2·12) | <0·01 | 1·14 (0·95-1·38) | 0·159 | 1·03 (0·81-1·31) | 0·828 |
|  |  |  |  |  |  |  |  |  |  |
| Swedish-born women | Never used HC |  |  | 1·00 (reference) |  | 1·00 (reference) |  | 1·00 (reference) |  |
|  | Current or recent use of HC | 5,069,374 | 1204 | 1·17 (1·08-1·28) | <0·01 | 1·33 (1·23-1·45) | <0·01 | 1·24 (1·11-1·38) | <0·01 |
|  | Used HC >6 months previously | 2,299,710 | 896 | 1·94 (1·78-2·12) | <0·01 | 1·26 (1·15-1·38) | <0·01 | 1·23 (1·10-1·38) | <0·01 |
|  |  |  |  |  |  |  |  |  |  |
| Nulliparous women | Never used HC |  |  | 1·00 (reference) |  | 1·00 (reference) |  | 1·00 (reference) |  |
|  | Current or recent use of HC | 3,118,035 | 349 | 0·90 (0·78-1·03) | 0·132 | 1·60 (1·38-1·86) | <0·01 | N/A |  |
|  | Used HC >6 months previously | 1,150,495 | 258 | 1·80 (1·54-2·10) | <0·01 | 1·27 (1·09-1·48) | 0·003 | N/A |  |
|  |  |  |  |  |  |  |  |  |  |
| Women < 35 years | Never used HC |  |  | 1·00 (reference) |  | 1·00 (reference) |  | 1·00 (reference) |  |
|  | Current or recent use of HC | 4,665,004 | 462 | 0·95 (0·84-1·08) | 0·461 | 1·31 (1·15-1·49) | <0·01 | 1·10 (0·92-1·31) | 0·284 |
|  | Used HC >6 months previously | 1,929,474 | 337 | 1·70 (1·48-1·94) | <0·01 | 1·22 (1·07-1·40) | 0·004 | 1·17 (0·98-1·41) | 0·089 |
|  |  |  |  |  |  |  |  |  |  |
| Women < 20 years | Never used HC |  |  | 1·00 (reference) |  | 1·00 (reference) |  | 1·00 (reference) |  |
|  | Current or recent use of HC | 1,233,365 | 13 | 4.03 (1.33-12.21) | 0.014 | 1.00 (0.14-6.90) | 1.00 | n.c. |  |
|  | Used HC >6 months previously | 154,524 | 4 | 1.47 (0.70-3.08) | 0.31 | 0.13 (0.01-2.10) | 0.15 | n.c. |  |

^a^ Adjusted for age at start of each exposure, level of education, place of birth, age at first full-term pregnancy, number of children, having received ovulation stimulating treatment and any diagnosis of infertility, polycystic ovarian syndrome, or endometriosis.

^b^ Adjusted for the same covariates as Model 1, including for body-mass index (BMI) and smoking (available for parous women only; 37% of the study population).
