# Supplementary material for: Hormonal contraception and risk of breast cancer and breast cancer in situ among Swedish women 15–34 years of age: A nationwide register-based study
Source: Lancet Reg Health Eur. 2022 Jul 29;21:100470. doi: 10.1016/j.lanepe.2022.100470 (PMC9340531; doi:10.1016/j.lanepe.2022.100470)
Supplement: Supplementary file 4 [file mmc4.doc]

# Data sharing statement

**Will individual participant data be available (including data dictionaries)?**

No

**What data in particular will be shared?**

Not applicable.

**What other documents will be available?**

Study protocol will be available with publication.

**When will data be available (start and end dates)?**

Not applicable.

**With whom?**

Not applicable.

**For what types of analyses?**

Not applicable.

**By what mechanism will data be made available?**

Not applicable.

Uppsala, 2021-10-13

Jenny Niemeyer Hultstrand and

Inger Sundström Poromaa
